# Editorial: Spatial transcriptome and single-cell sequencing for exploring molecular mechanisms of neuroimmunity and discovering novel markers of neurological diseases

**DOI:** 10.3389/fneur.2025.1582019

**Published:** 2025-04-03

**Authors:** Bowen Wang, Yachen Shi, Chao Cheng, Jian Song, Yuanbo Pan

**Affiliations:** ^1^Department of Neurosurgery, The First Affiliated Hospital of Bengbu Medical University, Bengbu, China; ^2^Department of Neurology, The Affiliated Wuxi People's Hospital of Nanjing Medical University, Wuxi, China; ^3^Department of Neurosurgery, The Affiliated Wuxi People's Hospital of Nanjing Medical University, Wuxi, China; ^4^Institute of Cardiovascular Sciences, The People's Hospital of Guangxi Zhuang Autonomous Region & Guangxi Academy of Medical Sciences, Nanning, China; ^5^Department of Neurosurgery, Second Affiliated Hospital, School of Medicine, Zhejiang University, Hangzhou, China

**Keywords:** spatial transcriptome, single-cell sequencing, neurological diseases, neuroimmunity, molecular mechanisms

Neuroimmunity is a vital field of study that investigates the interactions between the nervous system and the immune system. In recent years, it has emerged as one of the most cutting-edge areas of research in the life sciences. This field not only elucidates the complex cellular composition and functional characteristics of the brain but also provides a foundation for advancing precision treatment strategies (1, 2). Neuroimmunity offers a novel perspective to dissect the pathogenesis of various neurological diseases. However, diagnosis and treatment of central nervous system (CNS) diseases remain elusive due to intricate pathological mechanisms and limited effective therapeutic options (3). Despite these challenges, traditional research methodologies concentrate primarily on histological, physiological, and population-level analyses. Unfortunately, these approaches are insufficient to explore the transcriptomic characteristics and associated pathological and immune mechanisms at the single-cell level. This limitation significantly hinders our understanding of the complex regulatory networks that govern neuroimmune interactions.

In recent years, breakthroughs in methodologies such as single-cell sequencing and spatial transcriptomics have revolutionized neuroimmunity research (4, 5). Single-cell sequencing facilitates the high-resolution analysis of gene expression profiles at the individual cell level, enabling the identification of distinct cell subsets and their heterogeneity (6, 7). Furthermore, spatial transcriptomics preserves the spatial integrity of samples while deepening our understanding of molecular mechanisms (8). These advanced techniques not only elucidate the complex regulatory networks within the neuroimmune system but also provide important tools for the discovery of novel molecular markers and the development of innovative therapeutic strategies (9).

Cai et al. investigated glioblastoma multiforme (GBM) and demonstrated a strong association between CRYAB protein expression and poor patient prognosis. Their knockout experiments confirmed that CRYAB promotes tumor proliferation and invasion and regulates the tumor immune microenvironment. However, these experiments were conducted *in vitro* without subsequent *in vivo* validation, raising questions about their reliability and necessitating further confirmation in animal models. In contrast, Xie et al. examined the pathogenesis of ischemic stroke (IS) using a multi-omics approach that integrated human peripheral blood transcriptome data with single-cell RNA sequencing from mouse MCAO models. They conducted simultaneous animal experiments for corroboration. This integrative strategy identified 14 key senescence-associated secretory phenotype (SASP)-related genes and implicated basophils as major immune participants in the disease process. However, the cross-platform data integration used in this study may introduce technical biases that require further refinement to fully address. Although Cai et al. and Xie et al. focused on different diseases, their methodologies shared common features. Both studies leveraged single-cell sequencing technologies combined with other omics datasets (e.g., RNA-Seq, microarray) and employed multiple analytical tools, for instance Seurat for single-cell analysis, Cox proportional hazards regression for survival analysis, and LASSO regularization for feature selection. These approaches enabled the identification of key molecular drivers and their roles in disease pathogenesis, thereby providing a robust theoretical foundation for the development of novel diagnostic markers and targeted therapeutic strategies.

This research emphasizes the potential of single-cell sequencing technology in dissecting complex diseases and highlights the critical role of the immune microenvironment in neurological disorders. Li, Zhang et al. conducted a comprehensive analysis of multi-omics datasets, such as genomic, single-cell transcriptomic, and epidemiological data, to investigate the association between aging and NK cell dysfunction in Alzheimer's disease (AD), identifying CHD6 as a potential key driver of AD pathogenesis. In addition, He et al. reviewed the application of single-cell sequencing and spatial transcriptomics in AD research, revealing the dynamic roles of microglia and astrocytes in disease progression and their spatial correlation with neuronal injury. The discovery of novel biomarkers, such as p-tau181 and NfL, also provided promising avenues for the early diagnosis of AD. These studies have shed light on the significance of immune cell dysfunction and neuroinflammatory mechanisms in neurological diseases. Moreover, the analysis of the dynamics of spinal cord injury (SCI) by Li, Zhai et al. revealed a dual role of immune cells: in the acute phase, they play a protective role by removing necrotic tissue and pathogens; however, excessive activation may exacerbate tissue damage and neuronal death, thereby impairing neural tissue repair and regeneration. In summary, these studies underscore the complexity of immunological mechanisms in neurological diseases and provide valuable insights for biomarker discovery and therapeutic development.

With the rapid advancement of sequencing technology, in addition to single-cell sequencing, there are multiple sequencing techniques (such as RNA sequencing, single-molecule real-time sequencing, nanopore sequencing, etc.). A significant number of researchers, leveraging comprehensive transcriptome analysis in conjunction with advanced sequencing technologies, can not only identify specific biomarkers for the accurate diagnosis and prognosis of diseases but also systematically assess treatment efficacy and predict patients' clinical outcomes (10–12). However, spatial transcriptomics and single-cell sequencing technologies still face challenges such as vast and complex data volumes, imperfect multimodal algorithms, and high costs (13). Looking ahead, fostering innovation to address technical challenges, integrating and optimizing different technologies to characterize alterations in the neuroimmune microenvironment, and translating these insights into clinical strategies remain core research priorities. Such advancements could redefine disease classification and offer innovative theranostic approaches for complex neurological diseases.

The studies discussed in this editorial underscore recent breakthroughs in the field of neuroimmunology and their implications for advancing our understanding of complex neurological diseases. Through the application of state-of-the-art methodologies, such as single-cell sequencing and spatial transcriptomics, researchers have not only uncovered the intricate interplay between the nervous system and the immune system but also shed new light on the mechanisms underlying nervous system disorders. These studies elucidate critical disease-related molecules and their functional roles in pathological processes by integrating single-cell sequencing with complementary omics datasets. These studies highlight the unique advantages of single-cell technologies in analyzing disease heterogeneity and tracking dynamic changes in the immune microenvironment, thereby paving the way for precision medicine. Furthermore, this editorial emphasizes the importance of fostering interdisciplinary approaches within neuroimmunology. Collectively, these studies represent key advances in addressing major challenges in neurological research and improving clinical outcomes.
